# Primary Care Physicians’ Attitudes and Beliefs towards Chronic Low Back Pain: An Asian Study

**DOI:** 10.1371/journal.pone.0117521

**Published:** 2015-01-30

**Authors:** Regina W. S. Sit, Benjamin H. K. Yip, Dicken C. C. Chan, Samuel Y. S. Wong

**Affiliations:** School of Public Health and Primary Care, Faculty of Medicine, The Chinese University of Hong Kong, Hong Kong, Hong Kong; Baylor College of Medicine, UNITED STATES

## Abstract

**Background:**

Chronic low back pain is a serious global health problem. There is substantial evidence that physicians’ attitudes towards and beliefs about chronic low back pain can influence their subsequent management of the condition.

**Objectives:**

(1) to evaluate the attitudes and beliefs towards chronic low back pain among primary care physicians in Asia; (2) to study the cultural differences and other factors that are associated with these attitudes and beliefs.

**Method:**

A cross sectional online survey was sent to primary care physicians who are members of the Hong Kong College of Family Physician (HKCFP). The Pain Attitudes and Beliefs Scale for Physiotherapist (PABS-PT) was used as the questionnaire to determine the biomedical and biopsychosocial orientation of the participants.

**Results:**

The mean Biomedical (BM) score was 34.8+/-6.1; the mean biopsychosocial (BPS) score was 35.6 (+/- 4.8). Both scores were higher than those of European doctors. Family medicine specialists had a lower biomedical score than General practitioners. Physicians working in the public sector tended to have low BM and low BPS scores; whereas physicians working in private practice tended to have high BM and high BPS scores.

**Conclusion:**

The lack of concordance in the pain explanatory models used by private and public sector may have a detrimental effect on patients who are under the care of both parties. The uncertain treatment orientation may have a negative influence on patients’ attitudes and beliefs, thus contributing to the tension and, perhaps, even ailing mental state of a person with chronic LBP.

## Introduction

Chronic low back pain (LBP) is a serious global public health problem. In fact, it is considered to be the leading cause of absence from work and limitation of activity all over the world [1]. It is defined as pain or discomfort between the costal margins and the gluteal folds, with or without leg pain, which lasts for more than 12 weeks [2]. Nonspecific low back pain, defined as back pain “for which it is not possible to identify a specific cause”, account for more than 90% of chronic low back pain and a huge part of the daily workload in general practice [3]. A systematic review estimated that the global point prevalence of non-specific low back pain was approximately 11.9 ± 2.0% [4]. Chronic low back pain is a serious and costly health condition, with direct and indirect expenses that place a heavy financial burden upon health care systems [5]. Chronic low back pain condition has been estimated to cost 2% of the gross domestic product in developed countries [6, 7].

Despite LBP’s negative impact and increasing prevalence, patients with this condition are regularly ignored while their complaints are often misunderstood by health care providers. Consequently, they do not receive timely or effective treatment [8, 9]. This issue poses considerable challenges and frustrations for health care providers and engenders mistrust among patients [10]. Physicians’ attitudes and beliefs regarding low back pain could potentially influence their patients’ attitudes and beliefs. Indeed, ample evidence demonstrates that the clinicians’ attitudes and beliefs regarding low back pain seem to affect the beliefs of their patients [11,12]. Physicians’ attitudes and beliefs also appeared to influence their recommendations regarding LBP patients’ activities and work [12–14].

It is now well established that the biomedical model falls short in explaining and treating chronic LBP. A biomedical model refers to a biomechanical explanation of a disease, where disability and pain are a consequence of a specific pathology [14]. A recent systematic review also showed that a biomedical orientation has a negative association with patient education, adherence to treatment guidelines, and reported work and activity recommendations [12]. In contrast, increasing evidence supports the use of the biopsychosocial model in the management of chronic LBP [15–18], which emphasizes the role of psychological and social factors in the development and maintenance of complaints.

Currently, no information is available on the attitudes towards and beliefs about chronic low back pain of primary care physicians in Asia. Since musculoskeletal complaints, to which LBP contributes most, is the second most common reason for consulting a doctor and constitutes up to 10–20% of primary care consultations [19]. The evaluation of attitudes and beliefs will give a better understanding of the current adopted pain explanatory model as well as its possible impact on the management of chronic LBP.

The primary aim of this study was to evaluate the attitudes and beliefs towards chronic low back among primary care physicians in Asia. The secondary aims were to evaluate the cultural differences and the factors that are associated with these attitudes and beliefs.

## Methods

### Design and setting

We conducted a cross sectional online survey among primary care physicians in Hong Kong between October 2013 and March 2014. Ethics approval for the study was obtained from the Survey and Behavioral Research Ethics Committee (SBREC), the Chinese University of Hong Kong. The study followed the Declaration of Helsinki.

Doctors eligible for the survey were members of the Hong Kong College of Family Physicians (HKCFP, N = 1638). An online questionnaire with a cover letter was sent via email to all members registered with the HKCPF. One point of Continuous Medical Education (CME) score was awarded for every questionnaire submitted. A reminder was sent to the non-respondents 4 weeks later. We defined chronic low pain as pain or discomfort between the costal margins and the gluteal folds, with or without leg pain, which lasts for more than 12 weeks [2].

### Measurements

The Pain Attitudes and Beliefs Scale for Physiotherapist (PABS-PT) was originally developed for physiotherapists, but more recently has been applied to a cohort of general practitioners in the Netherland, the UK, Brazil and Ireland [20–23]. It is a self reported measure that discriminates between a biomedical and a biopsychosocial orientation regarding the management of chronic low back pain. The scale was developed by expert validation and analysis of 36 items extracted from four different health-related questionnaires in 2003 [14]. The original 20 item PABS-PT was further validated by Houben et al. in 2005 [20], and this resulted in a 19 item, highly-rated tool for the assessment of health care providers’ attitudes and beliefs [24]. Each item is scored on a six point Likert scale that ranges from totally disagree (score 1) to totally agree (score 6). A higher score on each subscale indicates a stronger biomedical or behavior treatment orientation, respectively. A cut off point signifying a high or low score has not been established by the developers of the questionnaires. Gender, age, years of practice, type of practice (private or public), postgraduate training, clinical interests and personal experience of LBP were collected.

The participants were divided into four subgroups for further analysis using the scatter plot with biomedical (BM) score versus biopsychosocial (BPS) score. The four groups include (i) Group I: physicians with a low BM score and alow BPS score (ii) Group II: physicians with a low BM score and a high BPS score (iii) Group III: physicians with a high BM and a low BPS score and (iV) Group IV: physicians with a high BM and a high BPS score.

### Statistical analysis

We hypothesized that Hong Kong (HK) practitioners have higher mean scores of BM than practitioners in Western countries, who, on average have a mean score of 30 and SD is 6.0 [13, 21]. A sample size calculation based on this hypothesis indicated a sample of 119 respondents was required to detect a difference of 1.8 with type I error set at 0.05, power of 0.9 and Cohen’s to be 0.3.

We also hypothesized that physicians with family medicine training would be more likely to fall inot Group II; whereas physicians without any family medicine training would more likely to fall into Group III.

The mean score and standard deviation of each subscale were calculated according to the methods specified by the questionnaire developers [14]. A Pearson’s correlation coefficient was calculated between the two subscales to evaluate any interrelationship. Descriptive statistics were used to summarize the demographic data of the participants. Multiple linear regression and multinomial logistic regression were used to investigate the demographic characteristics that could be associated with the beliefs and attitudes measured in the scale.

## Results

Emails were sent to one thousand six hundred and thirty eight physicians, of which one hundred and fifty six responded to the questionnaire. The overall response rate was 9.52%. Of the respondents, seventeen were excluded due to missing data, with the remaining one hundred and thirty nine suitable for analysis. The final sample size (n = 139) can reflect the target population (N = 1638) precisely with a confidence level of 95% and a margin of error of 8%.

The demographic and professional characteristics, as well as the PABS of the respondents were summarized in Table 1. The distribution of private (45.3%) and public practice (54.7%) was similar; the mean BM score was 34.8+/-6.1 and the mean BPS score was 35.6 (+/- 4.8). The Pearson’s correlation coefficient (r = 0.025, p = 0.769) showed that it was not statistically significant between the two subscales.

**Table 1:** Descriptive result of all studied variables (n = 139).

	Mean±SD / Count	Range / Percent
Age	42.0±11.0	24–78
Gender		
F	55	39.6%
M	84	60.4%
Years of clinical practice	16.1±10.0	2–60
Sector		
Public	76	54.7%
Private	63	45.3%
Current qualification		
General practitioner	50	36.0%
Family medicine basic or higher trainee	32	23.0%
Family medicine specialist	57	41.0%
Special interest		
No special interest	42	30.2%
Psychiatry	18	12.9%
Musculoskeletal/ Sport medicine and Pain medicine	28	20.1%
Mixed interest and others	51	36.7%
Suffer from any kind of chronic low back pain in the past one year		
No	105	75.5%
Yes	34	24.5%
Biomedical score	34.8±6.1	19–51
Biopsychosocial score	35.6±4.8	23–50

The impact of demographic factors and professional characteristics on PABS were summarized in Table 2. Family medicine specialists had a lower biomedical score than General practitioners. Subjects who had mixed interest, which was defined as mixed interest in either Psychiatry, Pain Medicine, Musculoskeletal/Sport Medicine plus or minus other interests, were found to have a higher biomedical score than subjects who did not have any special interest. A personal experience of low back pain had no association with the scores.

**Table 2:** Association of all studied variables and the two scores.

	Biomedical score				Biopsychosocial score			
	r / mean±sd	p*	β	p^	r / mean±sd	p*	β	p^
Age	0.075	0.377	0.05	0.692	-0.107	0.208	0.08	0.443
Gender								
F	35.0±6.1	0.745	Ref		35.0±4.8	0.308	Ref	
M	34.6±6.1		-1.04	0.334	35.9±4.8		1.71	0.060
Years of clinical practice Sector	0.030	0.727	-0.09	0.495	-0.134	0.117	-0.20	0.067
Public	33.8±5.8	0.039	Ref		35.3±4.8	0.572	Ref	
Private	35.9±6.2		1.09	0.330	35.8±4.8		1.54	0.103
Current qualification								
General practitioner	36.6±6.9	0.013	Ref		35.3±5.4	0.797	Ref	
Family medicine basic or higher trainee	34.8±5.3		-2.01	0.166	35.4±4.3		0.15	0.899
Family medicine specialist	33.1±5.4		-2.69	0.029	35.9±4.6		1.26	0.222
Special interest								
No special interest	33.7±5.9	<0.001	Ref		35.7±4.9	0.556	Ref	
Psychiatry	31.3±5.1		-2.50	0.135	36.9±3.5		2.15	0.127
Musculoskeletal/ Sport medicine and Pain medicine	33.8±6.3		-0.06	0.969	35.5±4.6		0.36	0.766
Mixed interest and others	37.4±5.5		3.27	0.012	35.0±5.3		0.05	0.967
Suffer from any kind of chronic low back pain in the past one year								
No	34.6±6.1	0.621	Ref		35.7±4.3	0.596	Ref	0.390
Yes	35.2±6.2		0.22	0.850	35.1±6.3		-0.84	

* p-value of Pearson correlation for continuous variables, Two samples T-test or One way ANOVA for categorical variables where appropriate.

^ p-value of Multiple regression; β is regression coefficient.

**Fig 1:**
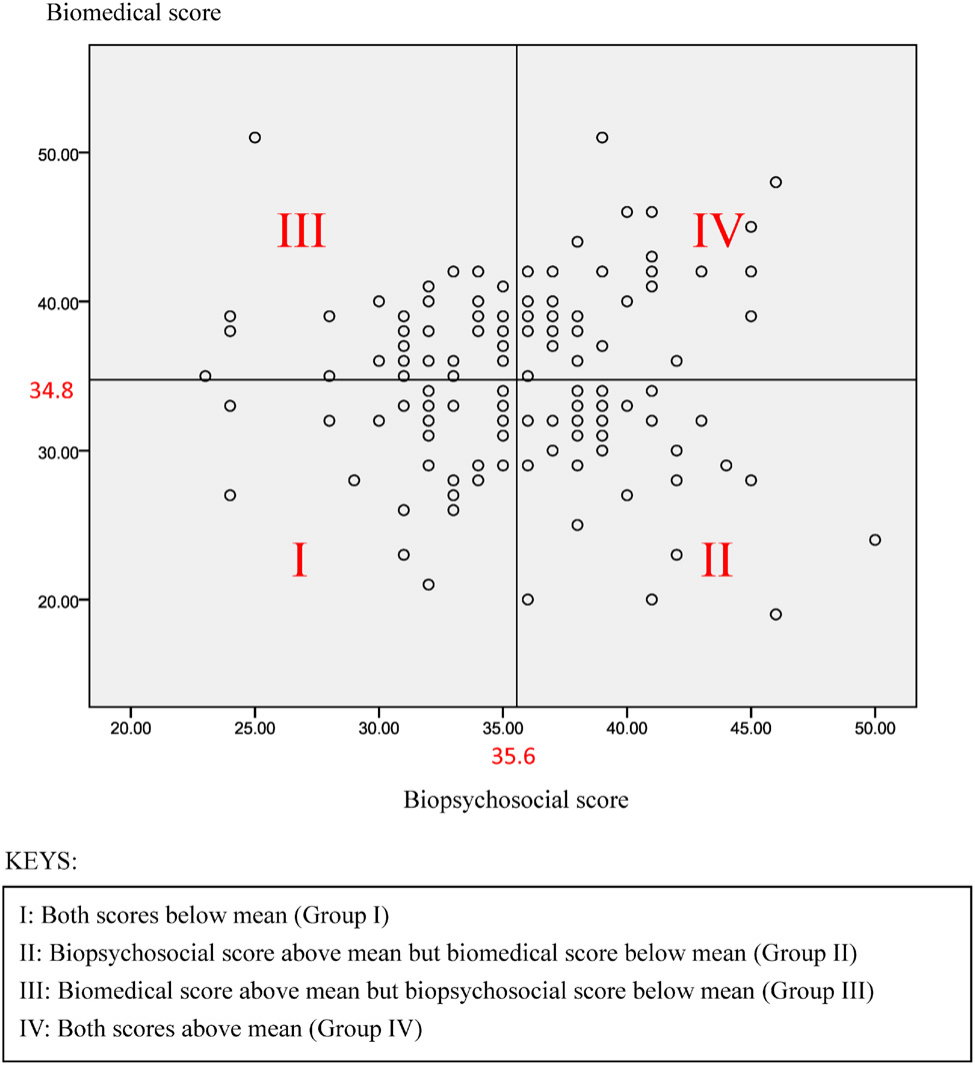
Scatter plot of biomedical score against biopsychosocial score (split into four parts according to means of two scores).

Participants were divided into four subgroups as shown in the scatter plot with BM score versus BPS score (Fig. 1). The sample size was similar in all four groups. Demographic and professional factors associated with different subgroups were summarized in Table 3. With all the studied variables, males tended to fall into Group II. Physicians working in the public sector tended to fall into Group I, whereas physicians working in private private with mixed interest tended to fall into Group IV (Table 4).

**Table 3:** Descriptive result of all studied variables by groups (Mean±SD / Count(%)).

	I (n = 37)	II (n = 31)	III (n = 35)	IV (n = 36)	p-value*
Age	42.3±12.3	40.1±8.8	44.1±12.6	41.4±9.7	0.501
Gender					
F	17(45.9%)	8(25.8%)	16(45.7%)	14(38.9%)	0.302
M	20(54.1%)	23(74.2%)	19(54.3%)	22(61.1%)	
Years of clinical practice Sector	17.2±12.4	14.3±8.9	17.0±9.7	15.4±8.2	0.595
Public	24(64.9%)	19(61.3%)	20(57.1%)	13(36.1%)	0.066
Private	13(35.1%)	12(38.7%)	15(42.9%)	23(63.9%)	
Current qualification					
General practitioner	10(27.0%)	10(32.3%)	14(40.0%)	16(44.4%)	0.224
Family medicine basic or higher trainee	6(16.2%)	7(22.6%)	8(22.9%)	11(30.6%)	
Family medicine specialist	21(56.8%)	14(45.2%)	13(37.1%)	9(25.0%)	
Special interest					
No special interest	14(37.8%)	9(29.0%)	10(28.6%)	9(25.0%)	**0.018** ^1^
Psychiatry	6(16.2%)	8(25.8%)	1(2.9%)	3(8.3%)	
Musculoskeletal/ Sport medicine and Pain medicine	10(27.0%)	7(22.6%)	5(14.3%)	6(16.7%)	
Mixed interest and others	7(18.9%)	7(22.6%)	19(54.3%)	18(50.0%)	
Suffer from any kind of chronic low back pain in the past one year					
No	30(81.1%)	23(74.2%)	25(71.4%)	27(75.0%)	0.808
Yes	7(18.9%)	8(25.8%)	10(28.6%)	9(25.0%)	

* p-value: One way ANOVA for continuous variables, Chi-squared test for categorical variables where appropriate.

^1^ Expected frequencies of some categories are too small, p-value is not reliable.

**Table 4:** Multinomial Logistic Regression model (Ref: Group I).

	OR (95% CI)		
	II	III	IV
Age	1.08 (0.88–1.32)	1.11 (0.92–1.35)	1.05 (0.86–1.28)
Gender			
F	Ref	Ref	Ref
M	**3.72 (1.13–12.21)**	0.81 (0.26–2.46)	1.20 (0.39–3.67)
Years of clinical practice Sector	0.87 (0.70–1.06)	0.89 (0.73–1.09)	0.90 (0.73–1.11)
Public	Ref	Ref	Ref
Private	1.56 (0.46–5.32)	1.06 (0.32–3.49)	**3.69 (1.14–12.01)**
Current qualification			
General practitioner	Ref	Ref	Ref
Family medicine basic or higher trainee	1.16 (0.23–5.93)	0.97 (0.20–4.66)	1.25 (0.27–5.75)
Family medicine specialist	1.01 (0.28–3.68)	0.59 (0.17–2.06)	0.45 (0.13–1.60)
Special interest			
No special interest	Ref	Ref	Ref
Psychiatry	3.68 (0.81–16.74)	0.23 (0.02–2.43)	1.17 (0.20–7.01)
Musculoskeletal/ Sport medicine and Pain medicine	1.34 (0.34–5.33)	0.59 (0.14–2.54)	1.24 (0.29–5.35)
Mixed interest and others	1.89 (0.44–8.11)	3.26 (0.87–12.17)	**4.13 (1.04–16.41)**
Suffer from any kind of chronic low back pain in the past one year			
No	Ref	Ref	Ref
Yes	1.57 (0.45–5.49)	1.67 (0.48–5.79)	1.33 (0.38–4.68)

## Discussion

### Main findings

This is the first survey of low back pain related attitudes and beliefs among a group of primary care physicians in Asia. The scores on the two subscales in the present study are very close. This may indicate an uncertainty among physicians regarding their treatment orientation [22].

Both BM and BPS scores are higher than those of a similar study conducted in the UK [21], which assessed the same domains with the same questionnaire items. This may be explained by the change in the undergraduate medical curriculum in our locality, which has involved the incorporation of more psychosocial elements into the traditional biomedical focused education [25, 26]. A direct comparison to the UK study with statistical analysis is not possible here, as we did not know the distribution of the data in the UK study.

Family medicine specialists have a lower biomedical score than General practitioners. They are not, however, necessarily associated with a high BPS score (group I and II). This may imply that current family medicine training may be good enough to prevent physicians from practicing with a predominantly biomedical approach. Whether a psychological model will be adopted may depend on multiple factors, such as time constraints, the practice setting and physicians’ own perceptions.

A strong contrast is noted between group I (low BM- low BPS) and group IV (high BM- high BPS). Physicians working in the public sector tended to fall into Group I; whereas physicians working in private sector tended to fall into Group IV. This can be explained by the fact that physicians in the public sector tend to minimize over-investigations and avoid abuse of public resources [27]. Therefore, they are more likely to adopt a less biomedical approach. Moreover, insufficent consultation time in a busy public setting and inadequate rapport with patients may potentially hinder them from adopting the biopsychosocial model. The opposite is true for private physicians, who are potentially more susceptible to medicolegal lawsuits and tend to over-investigate. They desire a medical diagnosis despite their understanding that psychosocial factors play a significant role in the pathogenesis of LBP [28].

Physicians in group IV are more likely to have mixed interests than those in group I. This is not surprising as physicians with knowledge and training in different aspects of LBP, starting from its pathogenesis to embracing the notion of psychosocial influences, will weigh both models as equally important.

Previous analysis showed that a biomedical and a biopsychosocial orientation were not two opposites on the same scale but, rather, that both factors were important in determining a physician’s orientation [14]. Until now, there have been no clear cut offs determined for “high” and “low” score on both BM and BPS subscales. There is also no evidence on the best combination of treatment orientation, although it is assumed that a low BM score and a high BPS score is preferred [15, 17]. Nevertheless, an individual physician’s worldview and orientation to the pain model will eventually influence his or her management decision on chronic LBP [12].

### Limitations

There are several limitations to the present study: Firstly, the low response rate in this survey (9.52%,) can lead to a high sampling bias and potentially limits the generalization of the study results. A “non-response” survey will be valuable in minimizing this bias. Secondly, the survey was completed via an online system which potentially excluded those who were not proficient computer users. Thirdly, the survey recruited only physicians who are members of the HKCFP, which, once again, limited its generalization. Fourthly, the study only looks into the treatment orientations of primary care physicians; it may not reflect their true clinical behaviors. Finally, the multiple comparisons conducted in this study are associated with a false positive correlation with 5% of the significance by chance. Meanwhile, there is still no universally accepted approach for dealing with the problem of multiple comparisons; it is an area of active research, into both the mathematical details and the broader epistemological questions.

### Implications for future research

The management of chronic LBP is moving towards a more integrated approach, especially in Asian countries, where Traditional Chinese Medicine and Acuputure are commonly practiced. Therefore, future studies can focus on comparing the attitudes, beliefs and clinical management of primary care physicians, orthopedic surgeons and Chinese Medicine Practitioners, with the concomitant evaluation of patients’ satisfaction. A new scope of these and how they relate to outcomes of patients is needed to generate the best model of care and to inform development of future implementation strategies.

## Conclusion

The study shows the diversity of the attitudes and beliefs of primary care physicians concerning the management of LBP. The lack of concordance in the pain explanatory models used by the private practice and public sector may have a detrimental effect on patients who are under the care of both parties. The uncertain treatment orientation may have a negative influence on patients’ attitudes and beliefs, thus contributing to the tension and ailing mentalhealthor constitution of a person with chronic LBP.
